# Supplementary material for: A Qualitative Exploration of UK Leisure Horse Owners’ Perceptions of Equine Wellbeing
Source: Animals (Basel). 2022 Oct 26;12(21):2937. doi: 10.3390/ani12212937 (PMC9654126; doi:10.3390/ani12212937)
Supplement: Supplementary file 1 [file animals-12-02937-s001.zip › Supplementary File S1 - Focus Group Schedule.pdf]

S1: Focus Group Schedule

Leisure Horse Owners and Equine Wellbeing Focus Group Schedule

Notes: workshops to be held online, via Zoom. Each workshop to have two hosts, who will swap activities as required. At any one time, one will chair and the other will ensure that content is covered, everyone has a chance to speak, etc.

Component 1: Welcome and introduction

- Overview of the project – "*we're interested in what horse wellbeing means to the people who care for horses. We would like to hear about your experiences and ideas in relation to areas such as horse happiness, health and quality of life. We are interested in everyone's views. What is said will be anonymised, and there are no right or wrong answers. As part of that, please remember to be respectful of others views and experiences, even if you disagree*".
- Session overview – "*we will have a mixture of questions and discussions, as well as activities as a group. Everyone will have a chance to speak when they want to, and it will be an informal discussion/conversation, so it's fine for you to build upon things others have said (as long as you are always respectful to them). If there are any parts of the discussion that you don't want to join in with, that is absolutely fine, you are under no pressure.*"
- Etiquette – "*we ask participants to raise a hand or make it visible on camera that they want to speak when possible.*" "*please be respectful of each other's' opinions and we ask that you keep the discussions that take place within the group private*"
- Recording – "*we will be recording the meeting, so that we can transcribe (type it up) afterwards. Any names you use, such as each other's' names, horse names, vets, and so on, will be altered in the transcription process so as to maintain everyone's anonymity*".
- Introduction/icebreaker – (the main host involved in this too)
  "*this is the hardest thing we will ask you to do – tell us about you and your horse in just thirty seconds!*"

Part 1 (around 40 mins):

- *Could you tell us about a specific time when you have thought about a/your horse's wellbeing (current horse or previous horse)?*

  Prompts when needed:
  - What prompted you to consider this? How do you manage a/your horse's wellbeing (other term depending on discussion above)?
  - Could you tell us about a time when you were concerned about a/your horse's wellbeing?
    Why were you concerned? (pick up on the meaning of words if they bring in quality of life and welfare) How did you respond?
  - Have there been times when you've had to compromise on wellbeing?
  - Do you think that the things important to give a horse a good level of wellbeing are different over the horse's life?
  - From your experiences, at what point would you consider compromised wellbeing to be unacceptable? How would you respond?

Part 2 (around 40 mins):

Participants are provided the terms of interest (*Quality of life, life worth living, welfare, wellbeing, good life, best life, happiness*) on virtual post it notes using the “Whiteboard” function of Zoom).

Participants told: “*Everyone has different views of these terms and what they mean – so there’s no wrong answers here. Looking at these terms, are there any that to you, that particularly stand out as being relevant to you and your horse?”*

Prompts if needed:

- Do you ever think about these terms in relation to your own horse(s)?
- Which term represents the best possible life?
- Which term represents the lowest possible quality of life?
- Do you find any terms to be associated with a particular life stage? or with any particular group of horses? What do these terms mean to you in that context?
- If we were discussing whether to put a horse through colic surgery, would you use the same terms? What if we were talking about keeping a horse in a stable for a few weeks over winter, when it hates being stabled?
