# Supplementary material for: A Qualitative Exploration of UK Leisure Horse Owners’ Perceptions of Equine Wellbeing
Source: Animals (Basel). 2022 Oct 26;12(21):2937. doi: 10.3390/ani12212937 (PMC9654126; doi:10.3390/ani12212937)
Supplement: Supplementary file 1 [file animals-12-02937-s001.zip › Supplementary item - Results of a content analysis of the terms of interest.pdf]

Results of a content analysis of the terms of interest.

Item	Number of instances	Meaning	Number of instances	Examples
Welfare	56	General sense of wellness	32	"*we've been monitoring our guys welfare. My wife's horse is coming back from a tendon injury that he did last summer, so we've been doing some rehab with him."*<br><br>*note: many participants used the terms "welfare" and "wellbeing" concurrently as in "*you're constantly thinking about the welfare and wellbeing of your horses*"
		An outcome (positive welfare)	3	*"But it is so worth it to see that welfare come out of him. It is that good welfare that he has got there in the care by somebody else. I am happy to make that compromise."*
		Basic acceptable standards provided to the horse (e.g. five freedoms)	9	*"every day I pass these two ponies in a field that isn't a great field. They have a field shelter, they don't get ridden. I know- I see people there every day, so they get water, but they don't look well cared for…they have fresh haylage, fresh water. But for me, I don't think it's a good life for the horses. But I do think that their welfare has been met. But is it the best life? No."*
		Minimum acceptable quality of life experienced by the horse	6	*"it was obviously in pain. It was obviously uncomfortable. That, to me, isn't welfare."*
		Provision	5	*"When we talk about welfare, for me that equates to human intervention, and if I am doing something to affect that horse's welfare in a way that restricts its natural way of living, that's welfare. I wouldn't say a horse that's on the Great Plains in America or a pony on Dartmoor or – unless I suppose we do have an impact because if we're farming land then we're encroaching on their natural habitat and perhaps restricting it in that way, in which case it then becomes a welfare issue."*
Wellbeing	22	Ambiguous – general sense of wellness, contentment, or health, Provisions to ensure wellbeing	19	*"I even come to the point of thinking, "Has he got the right rug on at the right time, is he going to be cold?" Which might sound irrational, but I think it definitely does, and that comes into their wellbeing."*

Results of a content analysis of the terms of interest.

		Basic acceptable standards provided to the horse (e.g. five freedoms)	2	*"those fundamentals that shouldn't change through a horse's life so that the basics for wellbeing, I read actually your summary and I've never heard it described as the three-Fs. But it's that, isn't it? It's from when a horse is born that it should have those fundamentals through its life"*
		Minimum acceptable quality of life experienced by the horse	1	*"welfare and wellbeing, it obviously worked out she had another three years fine, but if it ever happened again, I don't think we'd do that again, would we?"* [in relation to an elderly horse needing to be painfully hand-walked due to lymphangitis]
Quality of life	30	Positive experiences	7	*"If we go off to the beach, if we go off to a fun ride, she really enjoys it. She keeps up with all the others, even goes past some of the others. And I think that is a nice quality of life."*
		Minimum standards; is the life worth living?	19	*"we identified a severe underlying problem. And it was down to quality of life. This is a horse, it has got to be able to graze in a field, run and play, and skip and joy or whatever. And that horse would never do that."*
		Ambiguous – a sense of wellbeing	4	*"you start to put a measure against those points, and think, "Well, is he happy? Is he having a good quality of life?" His mental health may be there."*
Best life	23	An aspiration	8	*"I want them to have the best life that they can that I'm able to give them."*
		Optimum conditions or experience for the horse	12	*"a horse can have a good life but a horse's best life, it certainly wouldn't ever been ridden or has to do anything for humans…."*
Good life	10	An adequate, satisfactory experience or provision	10	*"I don't think it's a good life for the horses. But I do think that their welfare has been met. But is it the best life? No"*
Life worth Living	14	Minimum acceptable experience or provision	7	*"When you think about a life worth living, I think the horses that have the worst life are things like racehorses, they don't have a life worth living. They really don't. They don't have anything a horse needs, the top show jumpers and especially the top dressage horses, they don't have the life worth living at all."*

Results of a content analysis of the terms of interest.

		Positive experience	2	*"do I think my horse has a good mental health? And if he does, and he probably has a good life. He's probably happy, it's a life that's worth living.*
		Questioning moral judgements about horse experience	5	*"A life worth living. I don't think horses are that existential. (Laughter) When somebody says, "Oh, this horse is wasted on me," it is like they don't cry themselves to sleep because they are not winning championships. They don't have a concept of, "Is this worth it or not?"*
Happiness	65	Positive affect	6	*"I saw him being in a field, a massive field, with three other horses that my friend has, and he is just so happy. You can just tell. He is a different horse."*
		Contentment	6	*"I think if you want to know if your horse is happy, look for contentment. You know, are they chilled out when they're just roaming around having a munch?"*
		Absence of happiness	14	*"We finally found out she'd got a bartonella infection but it was almost like, people used to say to me she's really not well, she's not happy, you should have her put down."*
		horse's general acceptance of conditions; general wellbeing	39	*"he's quite happy to be turned out most of the time, and I am lucky enough to have access to plenty of grass and some stables at home."*
